# The Development of Global Genomic Surveillance of Respiratory Syncytial Virus: Insights From 25 Project Countries, 2019–2023

**DOI:** 10.1111/irv.70195

**Published:** 2026-03-15

**Authors:** Obadiah Kenji, Fernando Motta, Thomas Williams, Nicole Wolter, Ian G. Barr, Clyde Dapat, Maria Zambon, Lucy Mosscrop, Mei Shang, Sergejs Nikisins, Siddhivinayak Hirve, Wenqing Zhang, the WHO RSV Surveillance Group

**Affiliations:** ^1^ World Health Organization, Global Influenza Programme Geneva Switzerland; ^2^ Laboratório de Vírus Respiratórios Exantemáticos, Enterovírus e Emergências Virais, Instituto Oswaldo Cruz, FIOCRUZ Rio de Janeiro Brazil; ^3^ Child Life and Health University of Edinburgh Edinburgh UK; ^4^ Centre for Respiratory Diseases and Meningitis National Institute for Communicable Diseases of the National Health Laboratory Service Johannesburg South Africa; ^5^ School of Pathology, Faculty of Health Sciences University of the Witwatersrand Johannesburg South Africa; ^6^ WHO Collaborating Centre for Reference and Research on Influenza, VIDRL Peter Doherty Institute Melbourne Australia; ^7^ Virus Reference Department UK Health Security Agency London UK; ^8^ Imperial College London London UK

**Keywords:** capacity building, genomic sequencing, RSV, RSV project countries

## Abstract

**Background:**

From 2016 to 2018, the World Health Organization (WHO) initiated a global RSV surveillance pilot program in 14 countries, expanding to 25 countries from 2019 to 2023. As part of this, a sequencing program was introduced to improve the understanding of RSV global genetic diversity prior to and following the introduction of interventions such as passive immunization, vaccines, and antivirals.

**Methodology:**

All RSV sequence data submitted to GISAID by WHO project countries from January 1, 2019, to December 31, 2023, was analyzed to evaluate progress in sequencing, lineage distribution, and RSV fusion (F) protein diversity.

**Results:**

From 2019 to 2023, 44,571 RSV sequences were submitted to GISAID, including 61% RSV‐A and 39% RSV‐B, with 34% being whole‐genome sequences. WHO project countries contributed 13,280 sequences (30%), with submissions increasing from 158 in 2020 to 3716 in 2023. Median data submission time improved from 1116 days in 2020 to 206 days in 2023. The dominant lineage detected was a.d.1 (20%) for RSV‐A and B.D.4.1.1 (29%) for RSV‐B. The F protein sequences showed high amino acid conservation: 97% for RSV‐A and 96% for RSV‐B.

**Conclusion:**

Substantial progress has been made in RSV genomic sequencing capacities in WHO project countries as seen by the increased submissions and improved timeliness of sequence data. RSV exhibited co‐circulating lineages (RSV‐A and RSV‐B) with low F protein diversity. It is important to sustain and further strengthen RSV sequencing capacities in all WHO regions as part of the ongoing WHO Global Genomic Surveillance strategy.

## 1 Introduction

Respiratory syncytial virus (RSV) is a major cause of acute lower respiratory infections, particularly in children less than 5 years old [1], but also in other age groups (older adults) [2] and at‐risk groups (those with immunodeficiency, infants born preterm, or those with underlying respiratory or cardiovascular disease) [2, 3]. Globally, RSV is estimated to cause over 33 million cases of acute lower respiratory infections annually in children less than 5 years old, resulting in 3.6 million hospitalizations and approximately 101,400 deaths [4], with low‐ and middle‐income countries (LMICs) accounting for over 95% of severe cases [4, 5].

Effective global surveillance systems are essential for monitoring the evolution of RSV, tracking its spread across regions, and detecting genetic changes over time. Genomic surveillance is critical in identifying new variants that may pose public health risks, such as reduced vaccine effectiveness and altered performance of antiviral therapies and monoclonal antibodies. These efforts are important for guiding vaccine deployment and informing public health interventions to reduce RSV‐related morbidity and mortality globally [6, 7].

In 2016, the World Health Organization (WHO) initiated a global pilot program for RSV surveillance in response to RSV vaccines, immunotherapeutics, and antivirals reaching late‐stage clinical trials. Initially involving 14 countries, the program expanded in 2019 to include 25 countries under the Global Influenza Surveillance and Response System (GISRS) [8, 9, 10]. A sequencing component was then introduced to improve the understanding of RSV genetic diversity prior to and following the rollout of these new interventions. The initiative leveraged existing influenza surveillance frameworks to generate evidence to guide the introduction of RSV preventive measures and strengthen laboratory diagnostic and virological surveillance capacities [8].

The RSV fusion (F) protein plays a crucial role in viral infection and serves as the primary target for the majority of neutralizing activity in human serum [11]. Currently, licensed RSV vaccines (Arexvy and Abrysvo) and monoclonal antibodies (palivizumab, nirsevimab and more recently clesrovimab [12, 13]) specifically target the pre‐F protein, effectively reducing disease severity and preventing severe RSV infections in vulnerable populations [14, 15]. The RSV pre‐F protein contains six dominant antigenic sites (Ø, I, II, III, IV, and V) that are key targets for host neutralizing antibodies [11]. Among these, Sites Ø and V are recognized as major drivers of the antibody response in most individuals, with antibodies targeting this site demonstrating potent neutralizing activity [11, 16, 17]. Even though the F protein is highly conserved, continued genomic surveillance is critical to monitor the emergence and prevalence of potential resistance mutations that may affect the therapeutic efficacy of monoclonal antibodies or vaccines.

The COVID‐19 pandemic highlighted the feasibility and value of integrated respiratory virus surveillance. Investments in molecular diagnostics and sequencing infrastructure during the pandemic significantly enhanced participating countries' genomic surveillance capabilities. Building on these advancements, WHO has advocated for sustainable, integrated RSV genomic surveillance as part of its broader global strategy for genomic surveillance of pathogens with epidemic or pandemic potential [18]. Recently, specific guidance on RSV sequencing in the GISRS network has been published, underscoring best practices and standards that inform our approach [19].

This manuscript presents insights from RSV genomic surveillance conducted across 25 RSV project countries between 2019 and 2023. By analyzing genomic data available in the Global Initiative on Sharing All Influenza Data (GISAID), we highlight advancements in genomic surveillance, temporal distribution of RSV lineages, and the genetic diversity of the RSV F protein during this period.

## 2 Materials and Methods

### 2.1 Study Design and Data Sources

This study analyzed RSV sequence data available in GISAID between January 2019 and December 2023. The data were sourced from 25 RSV project countries across the six WHO regions. Table lists the countries in each WHO region and denotes which of these were RSV project countries. In addition to the 25 project countries, we retrieved RSV sequence data from all other countries globally for 2019–2023. These data were used to calculate global totals and comparative metrics (e.g., the proportion of sequences originating from project countries vs. non‐project countries in each region).

Whole‐genome sequences (WGS), partial sequences (G and F genes), and accompanying metadata—including specimen collection and submission dates, as well as geographical location—were downloaded and analyzed where applicable. WGS was defined according to GISAID criteria: genomes with more than 14,900 nucleotides were considered complete, and were further classified as high coverage (less than 1% Ns [undefined bases]) or low coverage (greater than 5% Ns). GISAID is a major global public repository for RSV sequences, and all the RSV project countries primarily submitted their sequence data to this platform.

The sequence submission date (i.e., sequences submitted to GISAID between January 2019 and December 2023) was used as a filter to download sequences for analysis of surveillance outputs (counts of sequence contributions, trends, and timeliness). These data were downloaded in February 2024. For analyses of virus circulation (lineage distribution by time and phylogenetic analysis) and fusion protein mutation analysis, data were grouped by year of specimen collection to reflect the actual occurrence of cases. These data were downloaded in April 2024.

In our timeliness analysis, we focused on WGS because our primary interest was the turnaround time for sharing high‐quality, complete genomic data. Moreover, the cost of generating a whole genome sequence is now comparable to that of a partial genome sequence, but it offers additional information on viral evolution and allows for more precise lineage assignment [20].

### 2.2 Lineage Diversity and Prevalence

A total of 2037 RSV‐A and 2017 RSV‐B whole genome sequences were generated between January 1, 2019, and December 31, 2023 (filtered by collection date), and submitted by RSV project countries. These sequences were downloaded from GISAID on April 29, 2024 and manually curated to remove incomplete sequences (genome length < 14,900 nucleotides). They were then aligned using Geneious Prime software v 2025.0, with alignment mapping against reference samples: RSV‐A (Human orthopneumovirus strain A2, KT992094) [21] and RSV‐B (RSVB/ *Homo sapiens* /USA/92P‐337‐01/1992, KP258745), using the following settings: High Sensitivity, 25 iterations, randomly seeking the best matches, and a minimum mapping confidence of 99.9%. Lineages were classified using Goya's classification system [20], implemented on the online Nextclade platform, v3.5.0 (accessed on April 29, 2024). The percentage of lineages was calculated monthly and visualized using a stacked bar plot created with the ggplot library in R software Version 4.3.3.

### 2.3 Phylogenetic Analysis

To illustrate the temporal and geographic distribution of RSV lineages, phylogenetic analysis was performed. WGS from the project countries were downloaded from GISAID and aligned using MAFFT v.7.526 [22] and lineage assignment was performed using Nextclade Version 3.5.0 [23].

Sequences with genome length < 14,900 nucleotides, > 2000 Ns, > 8 ambiguous sites, > 150 private mutations, any frameshift mutations, or unexpected stop codons were removed from the analysis. After quality filtering, 1538 RSV‐A and 1233 RSV‐B sequences remained for phylogenetic reconstruction. Reference sequences (Table S2) representing major lineages as described by Goya et al. [20] were included as the backbone in the trees. Time‐resolved trees were constructed using the maximum likelihood method with a generalized‐time reversible (GTR) nucleotide substitution model as implemented in IQ‐TREE2 Version 2.3.3 [24] and the least‐square dating method [25]. Support for each node was calculated with 1000 replicates using the ultrafast bootstrap method (PMID: 29077904). Bootstrap values ≥ 90% are shown on the nodes of the trees. Trees were visualized using the ggtree 3.10.1 [26] package in R Version 4.3.3.

### 2.4 Fusion Protein Mutation Analysis

Sequences were downloaded using the same parameters used for phylogenetic analysis. For the fusion protein mutation analysis, only complete F sequences that were correctly translated were included. All WGS plus the complete F protein coding region fragments available in GISAID during the study period were utilized. The objective was to ensure high confidence in the analysis and in the results generated. Even with this strict approach, the analysis included the majority of available data, totaling 3147 and 2431 sequences for RSV‐A and RSV‐B, respectively. The sequences were aligned using Geneious Prime software, with the same parameters described in the lineage diversity and prevalence section above.

The genetic variability of the RSV F protein was analyzed across key antigenic sites (Sites Ø–V) as previously defined [17, 27, 28]. Amino acid substitutions were examined to identify patterns of genetic stability and variability across sequences. The degree of conservation was quantified using an R script, and residue‐level variability was documented. A residue was considered conserved if ≥ 95% of the sequences shared the same amino acid at that position. Among non‐conserved residues (< 95%), we further classified variability by defining variable residues as those with substitution frequencies of ≥ 1% and < 10%, and highly polymorphic residues as those with substitution frequencies of ≥ 10%. Residues in Sites Ø and IV, where the monoclonal antibodies nirsevimab and clesrovimab bind, were closely examined with a literature search carried out on the amino acid substitutions identified to investigate the potential impact of these substitutions on susceptibility to antibody neutralization.

### 2.5 Statistical Analysis

Descriptive statistics were used to quantify RSV sequence submissions, lineage prevalence and regional variability. Timeliness of sequence data submission was evaluated by calculating the median number of days between sample collection and data deposition into GISAID, with interquartile ranges (IQRs). Statistical results were visualized through bar graphs, line plots, phylogenetic trees, and geographic maps.

## 3 Results

### 3.1 WHO Regions and Project Country Contributions, Trends, and Timeliness of RSV Genomic Surveillance (2019–2023)

**FIGURE 1:**
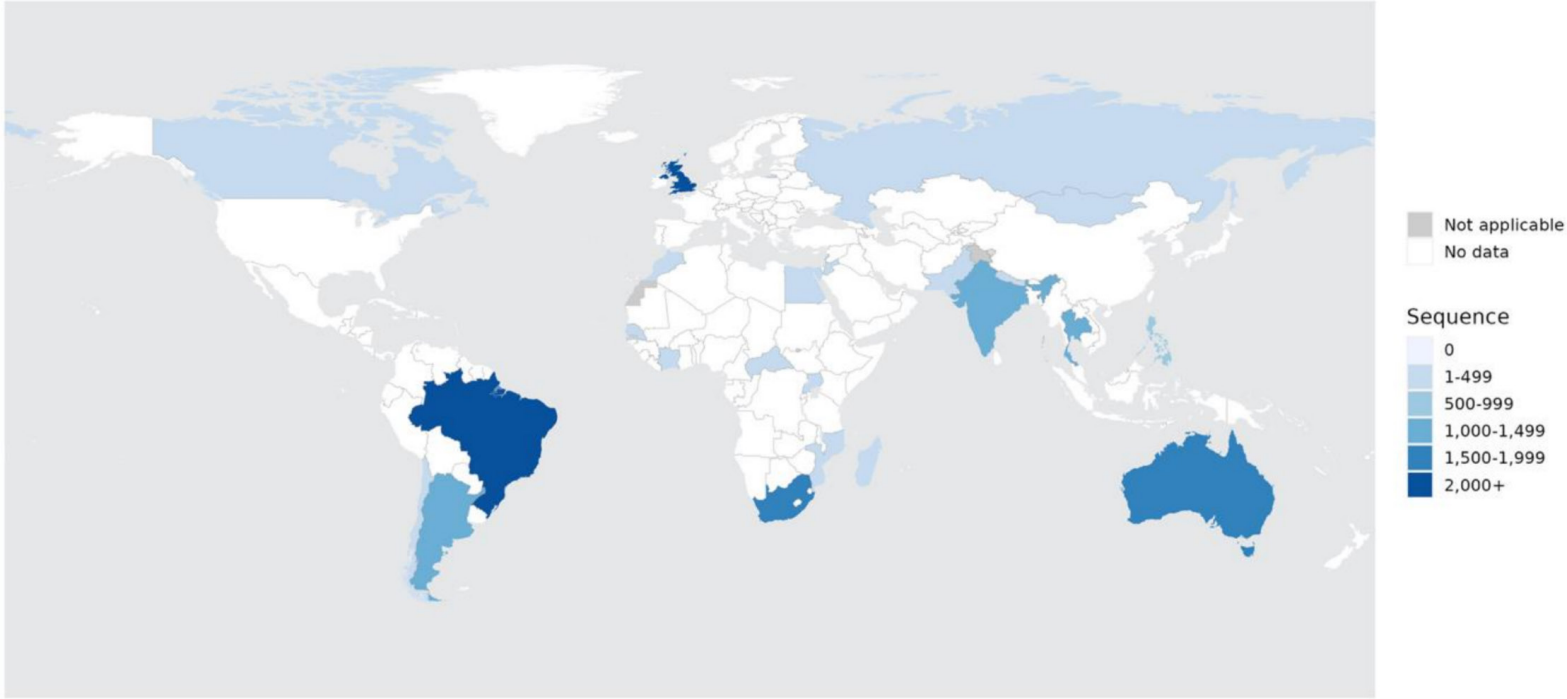
Map showing the RSV project countries and number of RSV sequences submitted to GISAID (2019–2023).

The RSV surveillance project involved 25 participating countries. These included 7 (28%) from the AFRO region, 6 (24%) from EMRO, 2 (8%) from EURO, 4 (16%) from AMRO, 3 (12%) from SEARO, and 3 (12%) from WPRO. Between 2019 and 2023, these countries made a significant contribution of (13,280/44,571; 30%) to global RSV genomic surveillance (Figure 1). The United Kingdom had the highest number of submissions (2249; 17%), followed by Brazil (2020; 15%), Australia (1864; 14%), and South Africa (1511; 11%). Other significant contributors included Argentina (1286; 10%), India (1002; 8%), Thailand (1060; 9%), and the Philippines (537; 4%).

**FIGURE 2:**
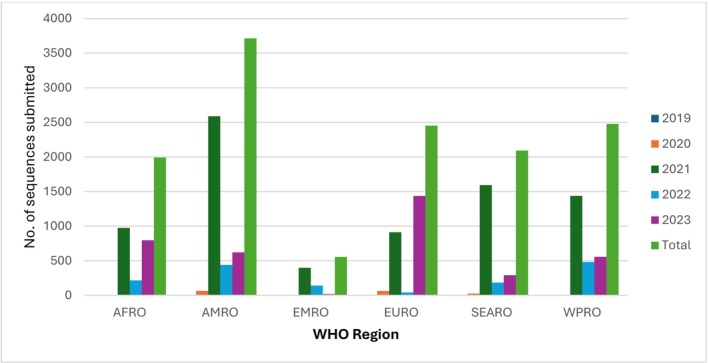
Number of RSV sequences (whole‐genome sequences and partial sequences) submitted by RSV project countries per WHO region by year, 2019–2023 (*N* = 13,280).

**FIGURE 3:**
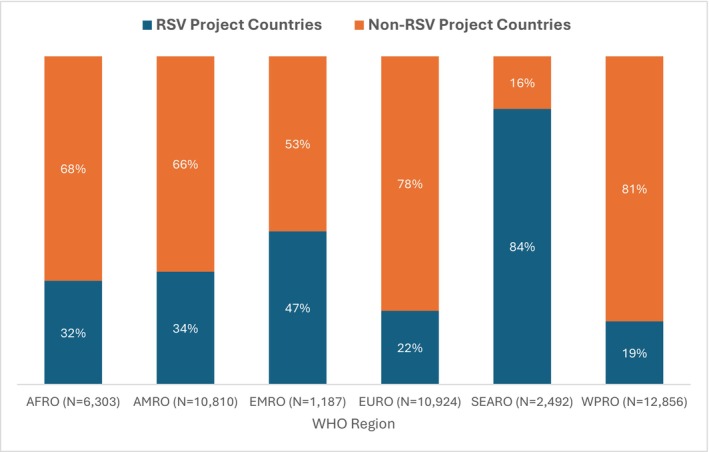
Percentage of RSV sequences (whole‐genome sequences and partial sequences) submitted to GISAID by project and non‐project countries, by WHO regions, 2019–2023 (*N* = 44,571).

The number of RSV sequence submissions by the project countries grew sharply over time (Figure 2). In 2020, only 158 sequences were submitted, but rose to 7903 in 2021—the highest recorded during the study period. After a decline to 1503 sequence submissions in 2022, the number rebounded in 2023 to 3716. The sequence submissions comprised 8154 (61%) RSV‐A, and 5126 (39%) RSV‐B sequences. Furthermore, 34% were WGS, while 66% were partial sequences. Figure 3 highlights regional disparities in contributions. In the SEARO region, RSV project countries accounted for 84% of sequence submissions by all countries in that region. By contrast, the RSV project countries provided 32% of the sequences in the AFRO region, 34% in AMRO, 22% in EURO, and 19% in WPRO. The EMRO region showed a more equitable distribution, with project countries contributing 47% of sequences.

Regarding total contributions from both RSV project and non‐project countries, WPRO accounted for 29% (12,856/43,571) of all RSV genomic data submissions. EMRO and SEARO contributed 3% (1187/43,571) and 6% (2492/43,571), respectively. AMRO submitted 25% (10,810/43,571) of the sequences, EURO submitted 25% (10,924/43,571), and AFRO submitted 14% (6303/43,571) (Figure 3).

The timeliness of data sharing improved over the study period. Table 1 illustrates a reduction in the time required to publicly share RSV genomic data. In RSV project countries, the median time from sample collection to data deposition decreased from 1116 days (IQR: 384 days) in 2020 to 206 days (IQR: 329 days) in 2023.

**TABLE 1:** Median days from sample collection to deposition of genomic data in GISAID (2020–2023).

Year	Number of WGS submitted		Median days to deposition	
	All countries	RSV project countries	All countries (IQR)	RSV project countries (IQR)
2020	51	50	1119 (384)	1116 (384)
2021	3916	1901	1973 (2975)	1231 (1701)
2022	1263	973	408 (812)	440 (929)
2023	4934	1655	399 (529)	206 (329)

### 3.2 Temporal and Geographical Distribution of RSV Lineages

**FIGURE 4:**
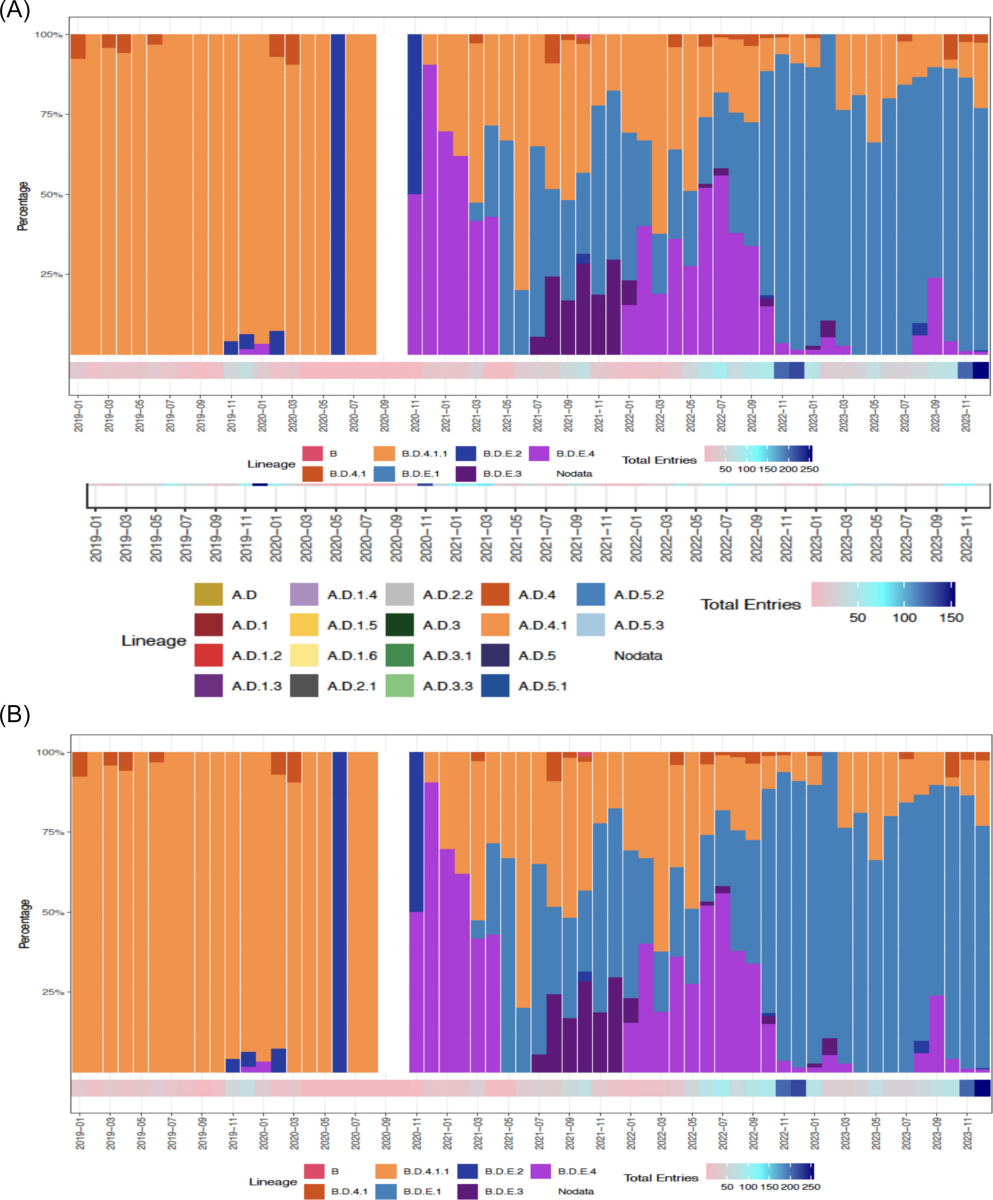
(A) RSV‐A lineages over time from WHO RSV project countries, 2019–2023 (*N* = 2017)*. *RSV‐A lineage analysis was based on a total of 2037 sequences comprising 24 lineages. Following a 1% prevalence threshold, 6 low‐frequency lineages (A, A.3, a.d.1.7, a.d.1.8, a.d.2.2.1, a.d.3.2) were excluded from the figure, resulting in 2017 sequences representing 18 lineages. (B) RSV‐B lineages over time from WHO RSV project countries, 2019–2023 (*N* = 2694)**. **RSV‐B lineage analysis was conducted using a total of 2694 sequences, after excluding 32 sequences due to the absence of collection month data or low confidence in the NextClade analysis.

**FIGURE 5:**
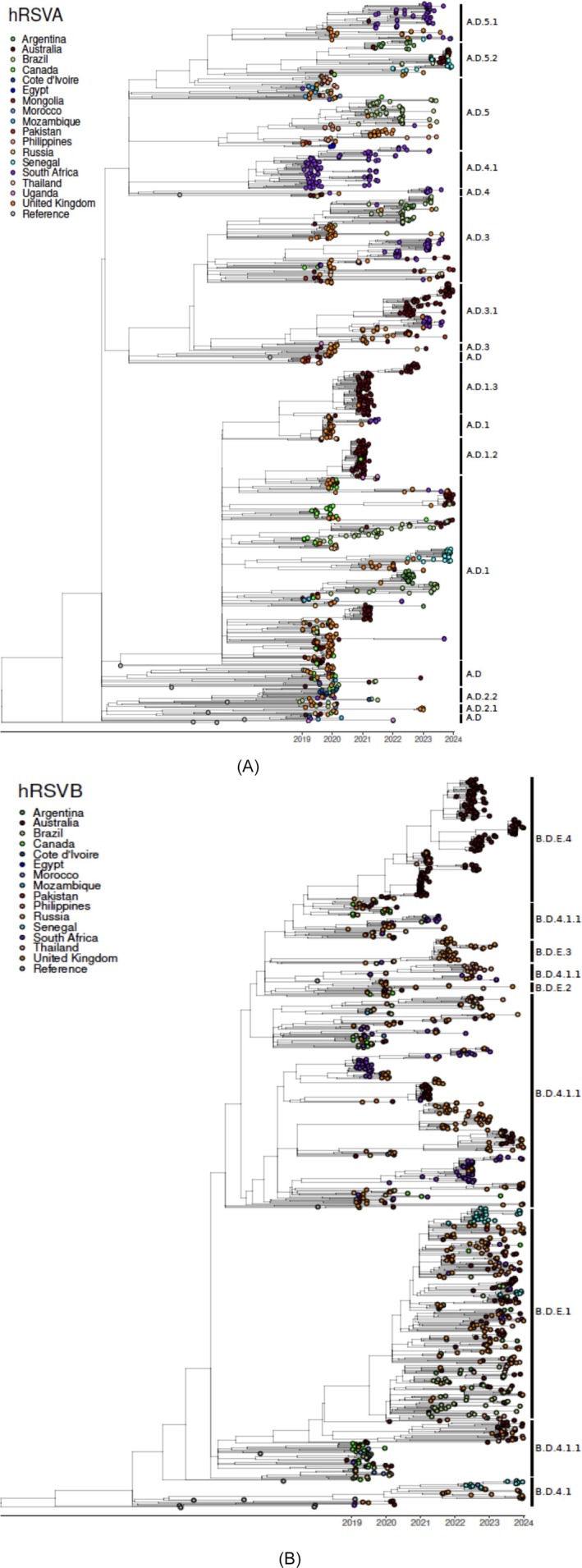
(A) Phylogenetic tree of RSV‐A WGS from WHO RSV project countries, 2019–2023 (*N* = 1538). (B) Phylogenetic tree of RSV‐B WGS from WHO RSV project countries, 2019–2023 (*N* = 1233).

Multiple co‐circulating RSV lineages were observed at any given time (Figures 4A,B, 5A,B, and S1–S5), though patterns differed between subgroups. The dominant lineage during the study period (2019–2023) was a.d.1 (20%) for RSV‐A. a.d.1 was the dominant lineage in 2019 (36%) and re‐emerged in 2021 (27%) after a.d.1.3 became predominant in 2020 (40%). In 2022, a.d.3.1 was the most prevalent RSV‐A lineage (22%), followed by a.d.5.1 in 2023 (19%). a.d.1 and its sub‐lineages were dominant in Asia and Oceania, with a.d.3.1 emerging later in this region. In Europe, a.d.1, a.d.3, and a.d.5.1 co‐circulated, alongside the appearance of a.d.2.1 and A.D.2.2. The African region displayed broader diversity, including a.d.4.1, a.d.5.1, a.d.5.2, and a.d.3.1, with continuing circulation of a.d.1. In the Americas, lineages a.d.1, a.d.5, and a.d.3 were dominant, with occasional detection of a.d.5.2 and A.D.2.2 in later years.

The most commonly identified RSV‐B lineage during the study period was B.D.4.1.1 (29%), but its prevalence decreased from 96% in 2019 to 69% in 2020 and 39% in 2021 (Figures 4B and 5B). This lineage maintained its global predominance across all regions (Figures S1–S5). However, in 2022, B.D.E.1 emerged as the most prevalent lineage (60%) and continued to be commonly identified in 2023, representing 79% of the sequences available for that year. In Asia and Oceania, B.D.4.1.1 persisted across multiple years, with B.D.E.1 emerging later. In Europe, B.D.4.1.1 after a period of high prevalence, was gradually replaced by emerging lineages like B.D.E.1 and B.D.E.3. Meanwhile in Africa, B.D.4.1.1 showed continuous circulation, with the emergence of B.D.E.1 in later years. In the Americas, B.D.4.1.1 decreased in frequency as B.D.E.1 became increasingly prevalent over time.

### 3.3 Mutation Analysis of the RSV Fusion Protein

**FIGURE 6:**
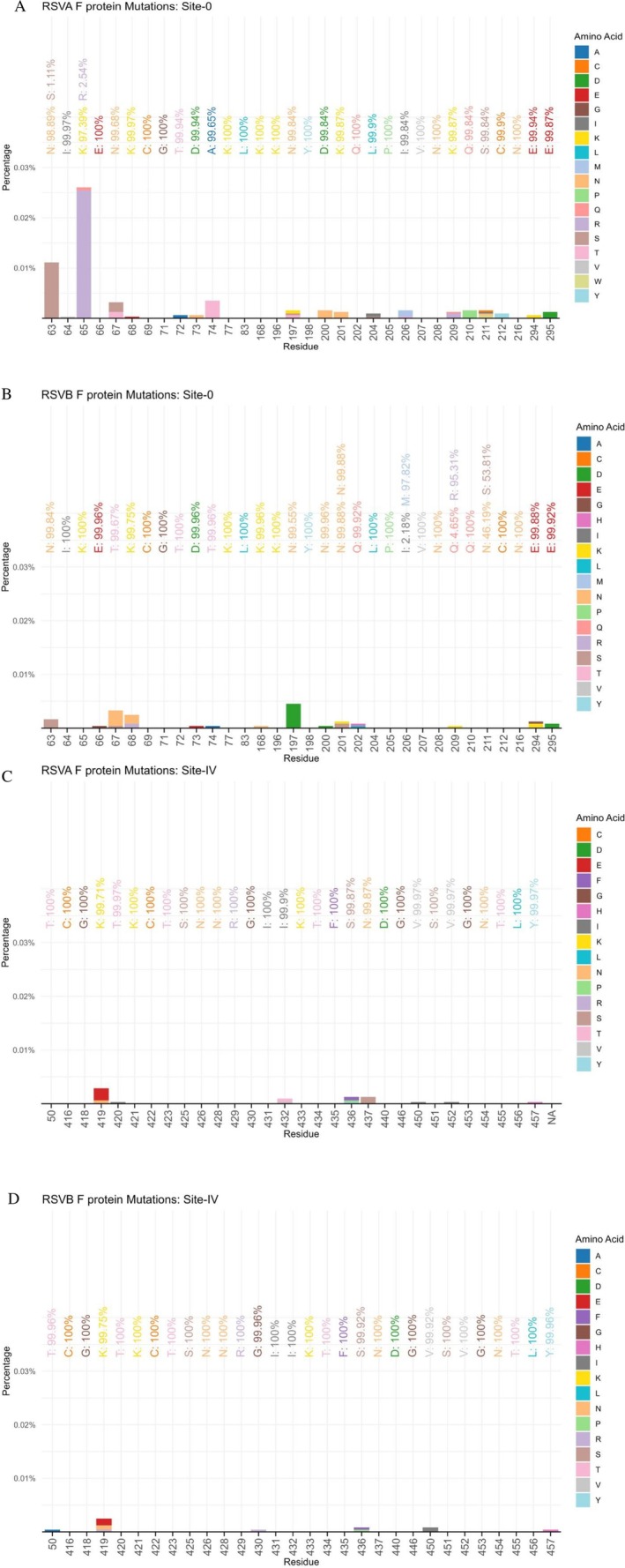
(A–D) F protein mutation analysis at antigenic sites Ø and IV among RSV sequences from project countries, 2019–2023 (*N* = 3147 RSV‐A; 2431 RSV‐B).

To assess the diversity of the F protein, complete F gene sequences on GISAID from 2019 to 2023 were analyzed across six antigenic sites (Ø and I to V). Overall, the diversity of RSV F protein sequences was low with 98% of residues being conserved across all the antigenic sites (Figures 6A–D, S6A, and S6B). For RSV‐A, 97% of the residues were conserved while 98% of the residues were conserved for RSV‐B. Unique conserved residues were detected at 63 of 64 positions (98%) in Site Ø, 36 of 40 positions (90%) in Site I, 39 of 40 positions (97%) in Site II, 98 of 100 positions (98%) in Site III, 60 of 60 positions (100%) in Site IV, and 40 of 40 positions (100%) in Site V. Sites IV and V exhibited complete conservation across all sequences, while Site I had the highest proportion of variable residues (4/40, 10%). Among antigenic sites, variable residues occurred at a frequency of less than 5%, with no highly polymorphic residues identified (≥ 10%).

## 4 Discussion

The global RSV genomic surveillance initiative represents a major advancement in understanding the genetic diversity of RSV, providing insights into global circulation, lineage diversity and F protein mutations using the GISRS platform.

### 4.1 Expansion of Genomic Surveillance Over Time

During the study period, 45,617 RSV sequences were submitted globally, with RSV project countries contributing 29% of these sequences. Notably, sequence submissions dropped sharply in 2020, consistent with the near‐total suppression of RSV circulation in that year due to COVID‐19 mitigation measures [29]. This decline was observed across regions and reflects both reduced RSV as a result of the application of non‐pharmaceutical interventions. By late 2021, as public health restrictions eased, RSV activity rebounded globally, which was mirrored in the increased number of sequences submitted in 2021 and subsequent years.

The dramatic increase in submissions, from 158 sequences in 2020 to 3716 in 2023 by the project countries, underscores the expanded genomic surveillance that this program has contributed. These submissions represent samples collected between 2019 and 2023, as well as retrospective specimens from earlier years that were subsequently uploaded to GISAID during the study period. Leading country contributors such as the United Kingdom, Brazil, and South Africa, highlight the impact of robust sequencing infrastructures in the post COVID‐19 pandemic era.

Despite these advances, disparities in the overall sequence contributions from project and non‐project countries persist among WHO regions. While WPRO accounted for 29% of submissions, SEARO (6%) and EMRO (3%) were underrepresented. These imbalances point to persistent challenges such as limited sequencing capacity, infrastructure gaps, and resource constraints in certain regions. These gaps are concerning, particularly as some of these regions are introducing RSV immunizations and vaccines without robust genomic surveillance programs. The absence of genomic data limits the ability to assess the global impact of interventions and detect emerging variants. Addressing these issues through sustained investment, workforce development, and improved data‐sharing mechanisms is crucial for equitable and more representative global RSV genomic surveillance [18].

Improved timeliness in sequence data submission—from a median of 1116 days in 2020 to 206 days in 2023—is a major achievement. According to the recently published WHO recommendations, RSV genomic data should be shared within about 3 months of the end of the RSV season. Thus, while the reduction of median submission time is a major improvement, there remains a gap compared to the recommended turnaround time. Timely data sharing is critical for real‐time viral evolution monitoring and public health responses. However, sustaining this progress requires continued investments in laboratory capacity, data‐sharing mechanisms, and international partnerships.

### 4.2 Patterns of Lineage Circulation

The genomic surveillance data from the 25 RSV project countries showed distinct lineage patterns for RSV‐A and RSV‐B. For RSV‐A, multiple co‐circulating lineages were observed at any given time, consistent with previous findings [20]. RSV‐A lineage a.d.1 and its sub‐lineages (a.d.1.2, a.d.1.3) demonstrated widespread persistence across regions and years. In contrast, RSV‐B showed less lineage diversity compared to RSV‐A, with fewer co‐circulating lineages and clearer evidence of sequential dominance. For example, lineage B.D.4.1.1 was dominant from 2019 to 2021. By 2022, lineage B.D.E.1 emerged as the dominant lineage and continued to increase in prevalence, reaching 79% in 2023, suggesting potential strain replacement. Notably, the lineage B.D.E.4 replaced these dominant lineages in the WPRO region from 2022 to 2023, in contrast to what was observed in the rest of the WHO regions.

Comparing RSV to influenza and SARS‐CoV‐2, there appear to be similarities to both, with RSV‐A showing co‐circulation of different lineages, similar to influenza [30], which undergoes frequent antigenic evolution due to antigenic drift, and RSV‐B showing evidence of lineage replacement similar to that seen for SARS‐CoV‐2 [31], which since emerging has demonstrated immune‐escape variants driven by natural infection and vaccine‐induced immunity [32]. For both influenza and SARS‐CoV‐2, these evolutionary changes have implications for vaccine design, with the need for periodic updates to vaccinations. At present, the vaccine implications of RSV evolution for vaccine design remain unclear, but our findings highlight divergent evolutionary dynamics between RSV subgroups and the need for sustained genomic surveillance to monitor viral evolution and guide global public health interventions until there is more clarity on the relevance of year‐to‐year viral variability on vaccine/immunization effectiveness.

### 4.3 Analysis of Potential Antigenic Evolution

Despite some potential antibody‐resistance associated substitutions being identified, the surveillance data revealed very low diversity in the RSV F protein over the study period, with 97% of amino acid positions being conserved, consistent with previous data [33, 34]. Antigenic sites Ø and V have been shown to elicit strong neutralizing antibody responses [11], and are typically prone to mutations; however, these sites were respectively 98% and 100% conserved. Site I, previously attributed to weakly or non‐neutralizing human monoclonal antibodies and considered lower in mutation potential [35] showed the highest number of mutations in our study, consistent with earlier findings [34]. For Site IV, the site at which clesrovimab binds, minimal variation was observed in both RSV‐A and RSV‐B subgroups with no resistance‐associated substitutions observed based on existing literature [36]. We also examined Site II, which contains the binding site for palivizumab and it was similarly well‐conserved. One RSV‐A sequence was identified with the resistance‐associated S275F substitution (palivizumab IC50 fold change of > 356) [37].

From the RSV‐A F genes analyzed in this study, there was some variation at position 63 of Site Ø with 1.1% of sequences containing a serine (S) instead of asparagine (N). This N63S substitution has been previously identified in nirsevimab‐breakthrough infections but not shown to confer resistance [38]. N63S has also been tested for its impact on nirsevimab susceptibility in RSV‐B where a 2.8‐fold increase in nirsevimab IC50 was demonstrated [37]. Residue 65 in Site Ø also had some variation with 2.5% of sequences containing an arginine (R) at the position in place of a lysine (K). This K65R substitution has been shown to confer a 3.9‐fold increase in nirsevimab IC50 [37]. Only one sample was found to contain the K68E mutation which has been linked to a 12.6‐fold increase in nirsevimab IC50.

From the RSV‐B F genes analyzed, position 206 of Site Ø had some variability with the majority of sequences having a methionine (M) but 2.2% contained isoleucine (I). Methionine at position 206 has been shown to confer a 5‐fold increased nirsevimab IC50 compared to isoleucine with I206M becoming prevalent in circulating RSV‐B strains from 2016 onwards [38]. Position 209 also has variable amino acids with 4.7% containing glutamine (Q) and the majority containing arginine (R). R at this position was shown to confer a marginal 0.5‐fold increase in nirsevimab IC50 [37] with the Q209R substitution appearing to now be fixed in circulating RSV‐B strains [38]. The other variable position seen in RSV‐B was 211 with 53.8% of sequences containing a serine (S) and 46.2% asparagine (N) at this position. Ser211Asn (S211N) substitution when present alongside Ile206Met (I206M) and Gln209Arg (Q209R) was associated with a 0.5‐fold change in nirsevimab IC50 [37]. Another study showed that S211N did not reduce the neutralization potency of nirsevimab [39]. Of interest is a K68N substitution that was found at a low prevalence of 0.2% but has been previously shown to confer a 29.9‐fold increase in nirsevimab IC50 [37].

These findings underscore the predominance of conserved residues across all F protein antigenic sites, reflecting the structural and functional importance of the F protein for RSV infectivity and immune targeting. However, these findings represent the RSV genomic landscape before the introduction of vaccines or monoclonal antibodies, meaning the observed stability may change under selective pressure, or due to drift. The data from the 25 RSV project countries provide an enhanced molecular baseline for tracking the frequency, geographic distribution, and evolutionary patterns of potential neutralization escape variants.

### 4.4 Incorporation of RSV Genomic Surveillance Into Global Influenza Surveillance Network

The expansion of genomic sequencing during the COVID‐19 pandemic provided a foundation for sustained respiratory virus surveillance. However, long‐term success depends on maintaining sequencing quality and capacity through strengthening workforce training, standardizing sequencing protocols, and implementing external quality assessment programs to ensure reliable data generation.

The successful incorporation of RSV genomic surveillance into the GISRS framework provides a model for multi‐pathogen surveillance. Capacity building during the COVID‐19 pandemic enhanced molecular diagnostics and sequencing infrastructure, which has been leveraged to incorporate and improve RSV surveillance. This integrated approach optimizes resources and strengthens preparedness for future respiratory epidemics and pandemics [18].

Future RSV genomic surveillance strategies should prioritize regions with limited sequencing capacity and low data representation relative to their population size or RSV disease burden. These investments would help ensure more balanced global data coverage and equitable monitoring of RSV evolution. In parallel, regions currently introducing RSV vaccines or monoclonal antibodies should receive targeted support to strengthen genomic monitoring of intervention impact. Comprehensive training programs and sustainable investments are necessary to sustain the core respiratory virus surveillance programs to have samples for sequencing. Integrating genomic, clinical, and epidemiological data will enhance understanding of RSV transmission and improve intervention evaluations. Continuous vaccine and therapeutic monitoring are critical to detect and mitigate the effect of potential escape variants. Strengthening global collaboration among public health agencies, research institutions, and industry stakeholders will foster a resilient RSV genomic surveillance framework and preparedness for future respiratory outbreaks. Future considerations for surveillance include ensuring that the diversity captured by WHO surveillance is representative profiles: this could include analyses to ensure that RSV WGS generated by, for example, research studies, are in keeping with what is seen in routine surveillance.

### 4.5 Limitations of Analysis Presented in This Manuscript

This study has several limitations. First, it relies on RSV sequence data available through GISAID, which may not capture all RSV cases globally and could introduce biases depending on each country's sequencing and data‐sharing practices. Second, there was uneven geographical representation in the dataset—some WHO regions were underrepresented in sequence submissions—which limits the generalizability of our findings. Also, our definition of “project country sequences” encompasses all data originating from a given country, whether generated by the National Influenza Centre or other laboratories. Third, most sequences analyzed were collected prior to the rollout of new RSV vaccines and monoclonal antibodies, meaning our results largely reflect a pre‐intervention viral genetic baseline. As such, ongoing genomic surveillance will be essential to observe and understand any viral evolutionary changes in response to these interventions moving forward. Finally, surveillance work is not designed to address scientific questions such as the ongoing circulation of RSV during the off‐season of viral circulation; these are likely to require higher levels of sequencing, likely embedded within a research project.

## 5 Conclusion

The WHO global RSV genomic surveillance initiative has substantially improved our understanding of RSV genetic diversity, lineage dynamics, and intervention preparedness. These findings highlighted the co‐circulation of multiple RSV lineages and the stability of key antigenic sites in the F (fusion) protein. Increased RSV genomic data, coupled with improved timeliness and capacity‐building efforts, underscore the importance of international collaboration and integrated surveillance systems. It is important to sustain and further strengthen RSV sequencing capacities in all WHO regions as part of the WHO Global Genomic Surveillance strategy.

## Author Contributions

**Obadiah Kenji:** conceptualization, methodology, formal analysis, writing ‐ original draft, writing ‐ review and editing (equal). **Fernando Motta:** conceptualization, methodology, formal analysis, writing ‐ review and editing (equal). **Thomas Williams:** conceptualization, methodology, writing ‐ review and editing. **Nicole Wolter:** methodology, writing ‐ review and editing. **Ian G. Barr:** writing ‐ review and editing. **Clyde Dapat:** formal analysis, data curation, writing ‐ review and editing. **Maria Zambon:** writing ‐ review and editing. **Lucy Mosscrop:** methodology, writing ‐ review and editing. **Mei Shang:** writing ‐ review and editing. **Sergejs Nikisins:** writing ‐ review and editing. **Siddhivinayak Hirve:** funding acquisition, supervision, writing ‐ review and editing. **Wenqing Zhang:** funding acquisition, writing ‐ review and editing. **WHO RSV Surveillance Group:** investigation, project administration, funding acquisition, data curation, methodology, writing ‐ review and editing.

## Funding

This work was supported by the Bill and Melinda Gates Foundation (78084).

## Conflicts of Interest

The authors declare no conflicts of interest.

## Supporting information


**Table S1:** Distribution of WHO member states by region.
**Table S2:** List of reference viruses in phylogenetic analysis.
**Figure S1A:** Temporal distribution of RSV lineages in the WHO African Region.
**Figure S1B:** Temporal distribution of RSV lineages in the WHO Eastern Mediterranean Region.
**Figure S1C:** Temporal distribution of RSV lineages in the WHO European Region.
**Figure S1D:** Temporal distribution of RSV lineages in the WHO Region of the Americas.
**Figure S1E:** Temporal distribution of RSV lineages in the WHO Western Pacific Region.
**Figure S1F:** Temporal distribution of RSV lineages in the WHO South‐East Asia Region.
**Figure S2A:** Phylogenetic tree of RSV‐A sequences from Asia and Oceania.
**Figure S2B:** Phylogenetic tree of RSV‐B sequences from Asia and Oceania.
**Figure S3A:** Phylogenetic tree of RSV‐A sequences from Europe.
**Figure S3B:** Phylogenetic tree of RSV‐B sequences from Europe.
**Figure S4A:** Phylogenetic tree of RSV‐A sequences from Africa.
**Figure S4B:** Phylogenetic tree of RSV‐B sequences from Africa.
**Figure S5A:** Phylogenetic tree of RSV‐A sequences from the Americas.
**Figure S5B:** Phylogenetic tree of RSV‐B sequences from the Americas.
**Figure S6A:** Mutation analysis of RSV‐A F protein.
**Figure S6B:** Mutation analysis of RSV‐B F protein.

## Data Availability

The data that support the findings of this study are openly available in GISAID at https://gisaid.org.
