# Correction to “The Development of Global Genomic Surveillance of Respiratory Syncytial Virus: Insights From 25 Project Countries, 2019–2023”

**DOI:** 10.1111/irv.70265

**Published:** 2026-05-18

O. Kenji , F. Motta , T. Williams , et al., “The Development of Global Genomic Surveillance of Respiratory Syncytial Virus: Insights From 25 Project Countries, 2019–2023,” Influenza and Other Respiratory Viruses 20, no. 3 (2026): e70195, 10.1111/irv.70195.41833533 PMC13098101

In Figure 4, Panel A and Panel B were duplicated. Panel A has been corrected. The correct Figure 4 is provided below.

**FIGURE 4:**
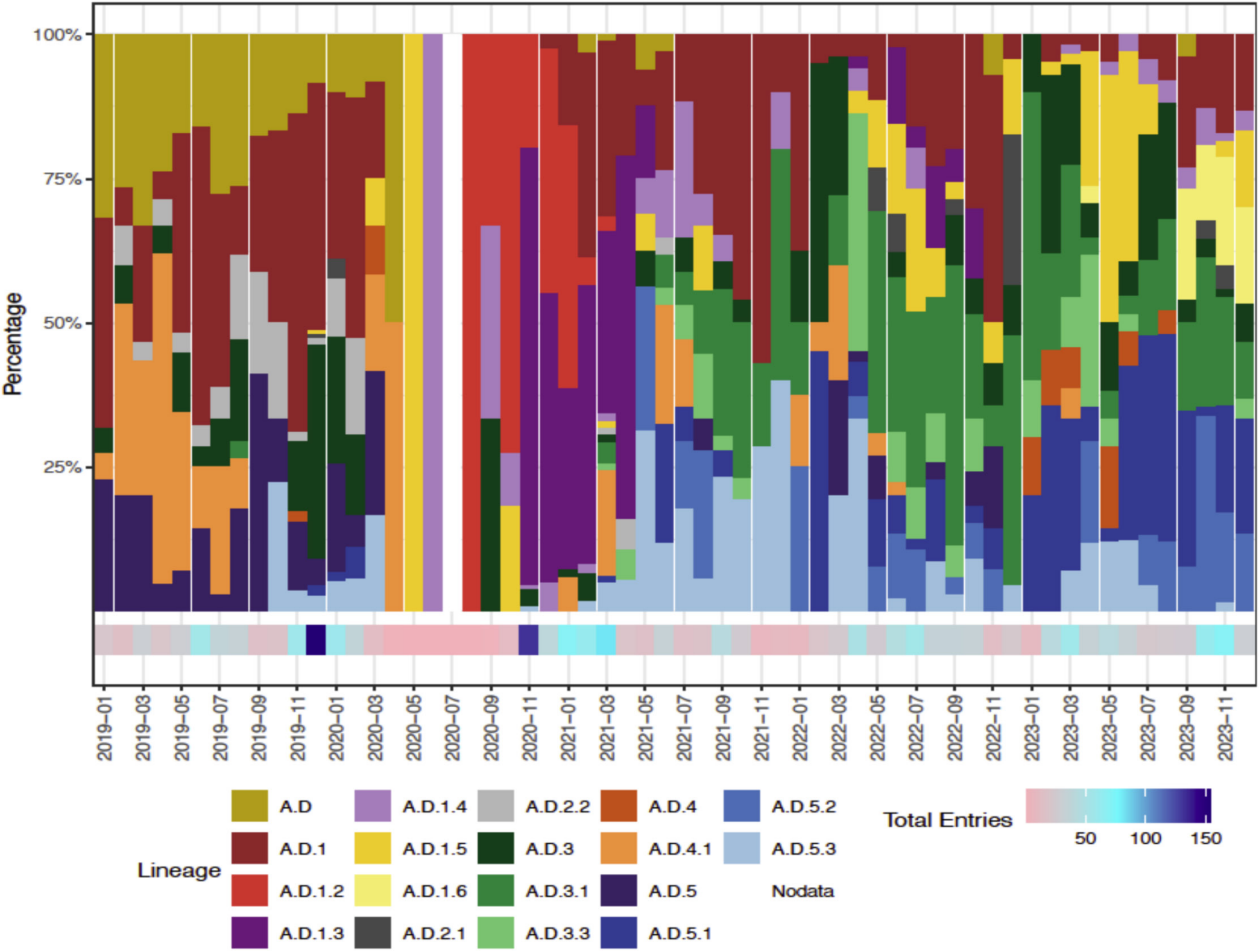
(A) RSV‐A lineages over time from WHO RSV project countries, 2019‐2023 (*N* = 2017)*. *RSV‐A lineage analysis was based on a total of 2037 sequences comprising 24 lineages. Following a 1% prevalence threshold, six low‐frequency lineages (A, A.e, A.D.1.7, A.D.1.8, A.D.2.2.1, A.D.3.2) were excluded from the figure, resulting in 2017 sequences representing 18 lineages.

We apologize for this error.

