# Supplementary material for: The Development of Global Genomic Surveillance of Respiratory Syncytial Virus: Insights From 25 Project Countries, 2019–2023
Source: Influenza Other Respir Viruses. 2026 Mar 15;20(3):e70195. doi: 10.1111/irv.70195 (PMC13098101; doi:10.1111/irv.70195)
Supplement: Supplementary file 1 — Table S1: Distribution of WHO member states by region. Table S2: List of reference viruses in phylogenetic analysis. Figure S1A: Temporal distribution of RSV lineages in the WHO African Region. Figure S1B: Temporal distribution of RSV lineages in the WHO Eastern Mediterranean Region. Figure S1C: Temporal distribution of RSV lineages in the WHO European Region. Figure S1D: Temporal distribution of RSV lineages in the WHO Region of the Americas. Figure S1E: Temporal distribution of RSV lineages in the WHO Western Pacific Region. Figure S1F: Temporal distribution of RSV lineages in the WHO South‐East Asia Region. Figure S2A: Phylogenetic tree of RSV‐A sequences from Asia and Oceania. Figure S2B: Phylogenetic tree of RSV‐B sequences from Asia and Oceania. Figure S3A: Phylogenetic tree of RSV‐A sequences from Europe. Figure S3B: Phylogenetic tree of RSV‐B sequences from Europe. Figure S4A: Phylogenetic tree of RSV‐A sequences from Africa. Figure S4B: Phylogenetic tree of RSV‐B sequences from Africa. Figure S5A: Phylogenetic tree of RSV‐A sequences from the Americas. Figure S5B: Phylogenetic tree of RSV‐B sequences from the Americas. Figure S6A: Mutation analysis of RSV‐A F protein. Figure S6B: Mutation analysis of RSV‐B F protein. [file IRV-20-e70195-s001.docx]

**Supplementary Table 1: Distribution of WHO Member States by Region**

| WHO Region | Countries |
| --- | --- |
| AFRO | Algeria, Angola, Benin, Botswana, Burkina Faso, Burundi, Cabo Verde, Cameroon, **Central African Republic*,** Chad, Comoros, Congo, **Côte d’Ivoire***, Democratic Republic of the Congo, Equatorial Guinea, Eritrea, Eswatini, Ethiopia, Gabon, Gambia, Ghana, Guinea, Guinea-Bissau, Kenya, Lesotho, Liberia, **Madagascar***, Malawi, Mali, Mauritania, Mauritius, **Mozambique***, Namibia, Niger, Nigeria, Rwanda, Sao Tome and Principe, **Senegal***, Seychelles, Sierra Leone, **South Africa***, South Sudan, Togo, **Uganda***, United Republic of Tanzania, Zambia, Zimbabwe |
| SEARO | Bangladesh, Bhutan, Democratic People’s Republic of Korea, **India***, Indonesia, Maldives, Myanmar, **Nepal***, Sri Lanka, **Thailand***, Timor-Leste |
| WPRO | **Australia***, Brunei Darussalam, Cambodia, China, Cook Islands, Fiji, Japan, Kiribati, Lao People’s Democratic Republic, Malaysia, Marshall Islands, Micronesia (Federated States of), **Mongolia***, Nauru, New Zealand, Niue, Palau, Papua New Guinea, **Philippines***, Republic of Korea, Samoa, Singapore, Solomon Islands, Tonga, Tuvalu, Vanuatu, Viet Nam |
| EMRO | Afghanistan, Bahrain, Djibouti, **Egypt***, Iran (Islamic Republic of), Iraq, **Jordan***, Kuwait, **Lebanon***, Libya, **Morocco***, Oman, **Pakistan***, **Qatar***, Saudi Arabia, Somalia, Sudan, Syrian Arab Republic, Tunisia, United Arab Emirates, Yemen |
| EURO | Albania, Andorra, Armenia, Austria, Azerbaijan, Belarus, Belgium, Bosnia and Herzegovina, Bulgaria, Croatia, Cyprus, Czechia, Denmark, Estonia, Finland, France, Georgia, Germany, Greece, Hungary, Iceland, Ireland, Israel, Italy, Kazakhstan, Kyrgyzstan, Latvia, Lithuania, Luxembourg, Malta, Monaco, Montenegro, Netherlands (Kingdom of the), North Macedonia, Norway, Poland, Portugal, Republic of Moldova, Romania, **Russian Federation***, San Marino, Serbia, Slovakia, Slovenia, Spain, Sweden, Switzerland, Tajikistan, Türkiye, Turkmenistan, Ukraine, **United Kingdom of Great Britain and Northern Ireland***, Uzbekistan |
| AMRO | Antigua and Barbuda, **Argentina***, Bahamas, Barbados, Belize, Bolivia (Plurinational State of), **Brazil***, **Canada***, **Chile***, Colombia, Costa Rica, Cuba, Dominica, Dominican Republic, Ecuador, El Salvador, Grenada, Guatemala, Guyana, Haiti, Honduras, Jamaica, Mexico, Nicaragua, Panama, Paraguay, Peru, Saint Kitts and Nevis, Saint Lucia, Saint Vincent and the Grenadines, Suriname, Trinidad and Tobago, United States of America, Uruguay, Venezuela (Bolivarian Republic of) |

*WHO RSV project countries, highlighted in **bold**.

**Supplementary Table 2. List of reference viruses in phylogenetic analysis.**

| Virus name | Accession | Collection date | Subtype |
| --- | --- | --- | --- |
| hRSV/A/Germany/MV-17-02326/2017 | EPI_ISL_17995645 | 2017-01-23 | A |
| hRSV/A/USA/un-UV-7H5/2011 | EPI_ISL_17673349 | 2011-01 | A |
| hRSV/A/Brazil/un-38566/2010 | EPI_ISL_15752035 | 2010 | A |
| hRSV/A/Kenya/Kilifi-1159-24-05/2013 | EPI_ISL_2595473 | 2013-05-24 | A |
| hRSV/A/Germany/SN-11-00953/2011 | EPI_ISL_17995630 | 2011-01-10 | A |
| hRSV/A/USA/ACRI-053/2016 | EPI_ISL_2583063 | 2016-03-07 | A |
| hRSV/A/Argentina/BA-HNRG-123/2015 | EPI_ISL_1074262 | 2015-05-27 | A |
| hRSV/A/United_Kingdom/DP-IM02-0033-V01/2018 | EPI_ISL_15753556 | 2018-11-30 | A |
| hRSV/A/Spain/un-DP-SE01A-0064-V02/2018 | EPI_ISL_15753425 | 2018-11-28 | A |
| hRSV/A/Netherlands/un-DP-UU03-0060-V02/2017 | EPI_ISL_15753349 | 2017-12-11 | A |
| hRSV/A/Argentina/BA-HNRG-270/2016 | EPI_ISL_1074141 | 2016-06-27 | A |
| hRSV/A/Nicaragua/NIC-IIIn-15-0256-01NT/2015 | EPI_ISL_2595691 | 2015-11-16 | A |
| hRSV/A/Kenya/KILIFI-WGS-1249-05-12/2014 | EPI_ISL_2578814 | 2014-12-05 | A |
| hRSV/A/Thailand/TH-B10806/2014 | EPI_ISL_2582296 | 2014-08-27 | A |
| hRSV/A/USA/TX-121301033/2012 | EPI_ISL_15752007 | 2012 | A |
| hRSV/B/USA/TH-10290/2012 | EPI_ISL_2585075 | 2012-10-16 | B |
| hRSV/B/Netherlands/un-DP-UU02-0005-V01/2017 | EPI_ISL_15753027 | 2017-11-17 | B |
| hRSV/B/USA/un-UV-7I6/2011 | EPI_ISL_17673362 | 2011-01 | B |
| hRSV/B/United_Kingdom/583/2013 | EPI_ISL_2577426 | 2013-12-18 | B |
| hRSV/B/Japan/B-Sendai-1077-16/2016 | EPI_ISL_2558899 | 2016-12-31 | B |
| hRSV/B/Argentina/BA-HNRG-206/2016 | EPI_ISL_1074181 | 2016-04-18 | B |
| hRSV/B/Australia/un-WIMR-79B/2017 | EPI_ISL_18090641 | 2017 | B |
| hRSV/B/China/CCMU-WZ17010/2017 | EPI_ISL_18482725 | 2017-12-22 | B |
| hRSV/B/Netherlands/un-DP-UU01A-0026-V02/2018 | EPI_ISL_15753202 | 2018-11-15 | B |
| hRSV/B/United_Kingdom/OX03-0187-V02/2018 | EPI_ISL_15753171 | 2018-11-01 | B |
| hRSV/B/Nicaragua/NIC-IIIn-15-0233-01NT/2015 | EPI_ISL_2577412 | 2015-11-05 | B |
| hRSV/B/Argentina/BA-HNRG-014/2014 | EPI_ISL_1074215 | 2014-05-20 | B |
| hRSV/B/United Kingdom/136/2014 | EPI_ISL_2577396 | 2014-01-07 | B |
| hRSV/B/Jordan/JOR-A0777/2013 | EPI_ISL_2577754 | 2013-03-25 | B |
| hRSV/B/United Kingdom/132/2012 | EPI_ISL_2544125 | 2012-01-06 | B |

**Supplementary Figure 1A: Temporal distribution of RSV lineages in the WHO African Region**

**Supplementary Figure 1B: Temporal distribution of RSV lineages in the WHO Eastern Mediterranean Region**

**Supplementary Figure 1C: Temporal distribution of RSV lineages in the WHO European Region**

**Supplementary Figure 1D: Temporal distribution of RSV lineages in the WHO Region of the Americas**

**Supplementary Figure 1E: Temporal distribution of RSV lineages in the WHO Western Pacific Region**

**Supplementary Figure 1F: Temporal distribution of RSV lineages in the WHO South-East Asia Region**


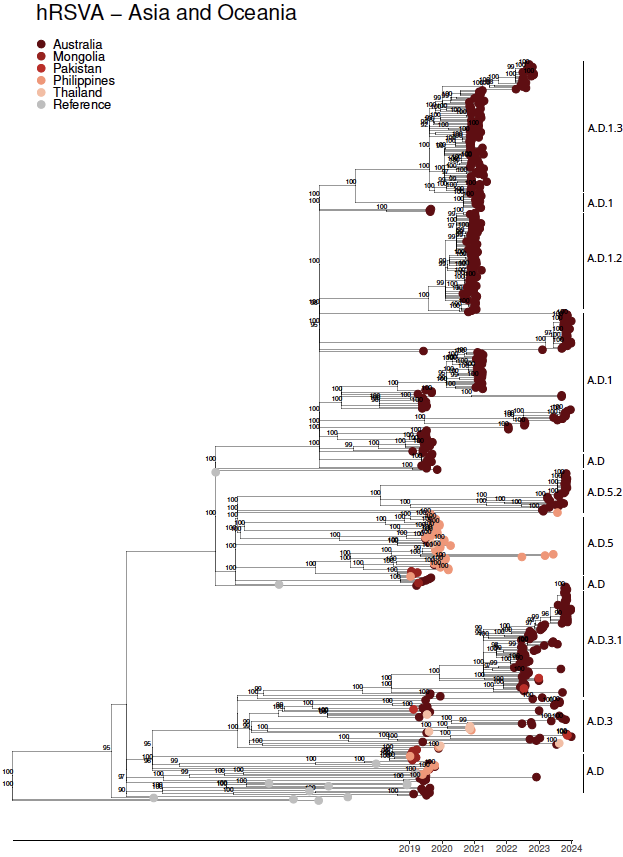


**Supplementary Figure 2A:** Phylogenetic tree of RSV-A sequences from Asia and Oceania


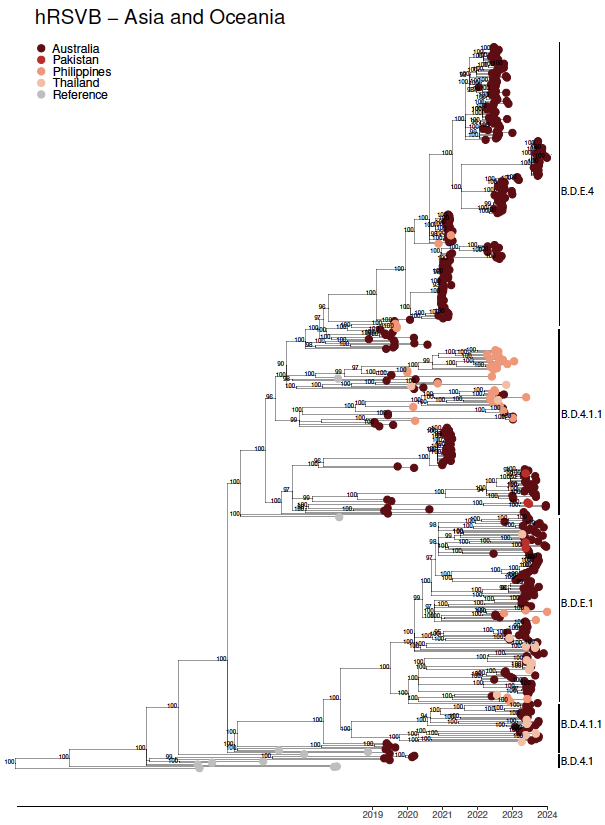


**Supplementary Figure 2B:** Phylogenetic tree of RSV-B sequences from Asia and Oceania


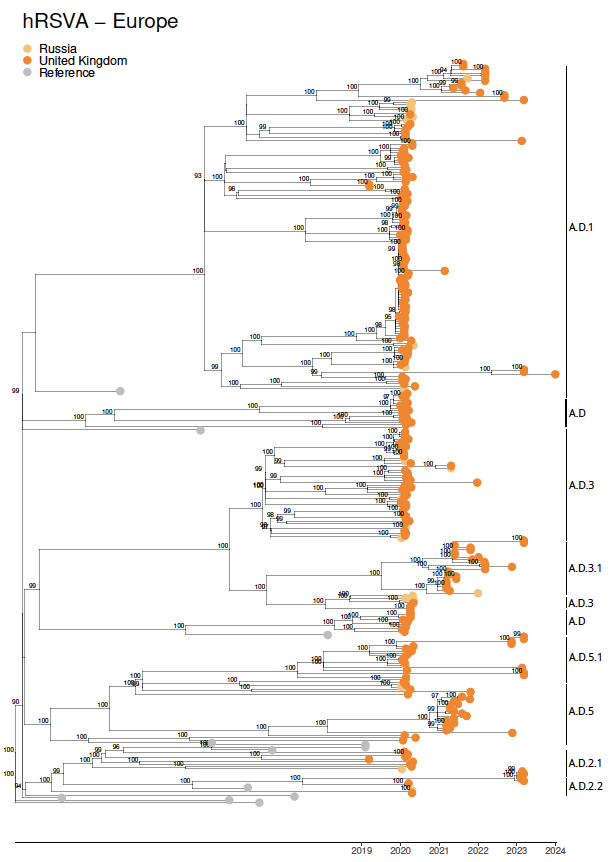


**Supplementary Figure 3A:** Phylogenetic tree of RSV-A sequences from Europe


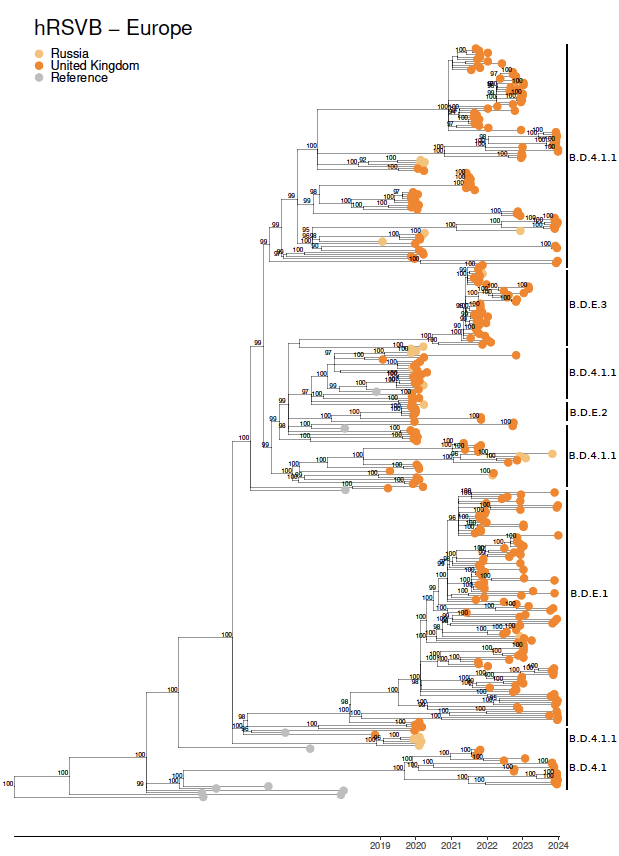


**Supplementary Figure 3B:** Phylogenetic tree of RSV-B sequences from Europe


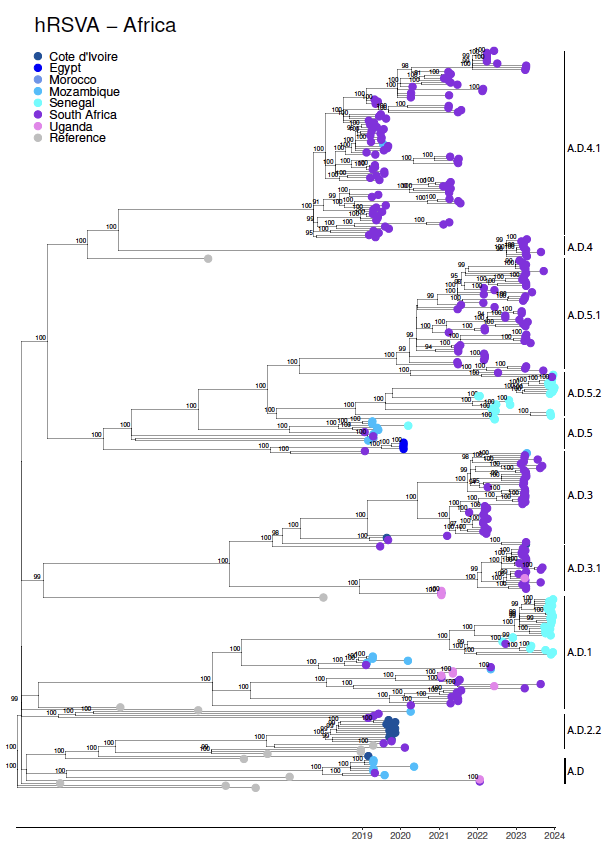


**Supplementary Figure 4A:** Phylogenetic tree of RSV-A sequences from Africa


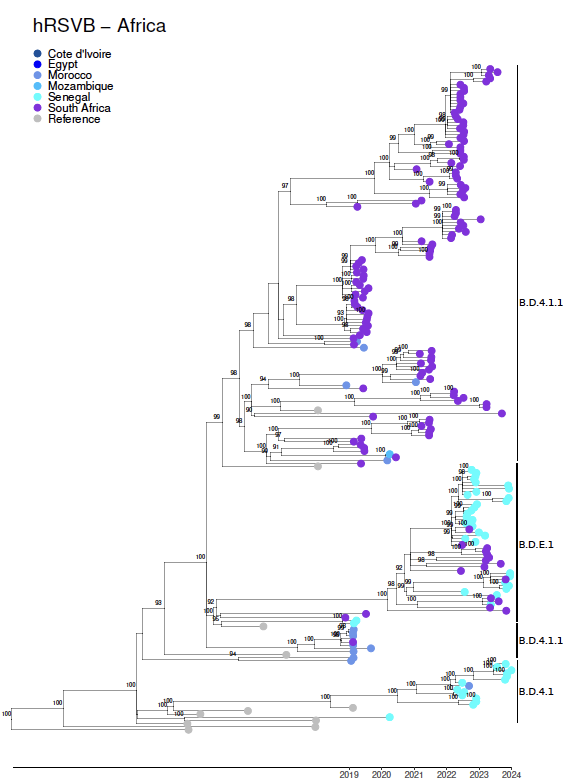


**Supplementary Figure 4B:** Phylogenetic tree of RSV-B sequences from Africa


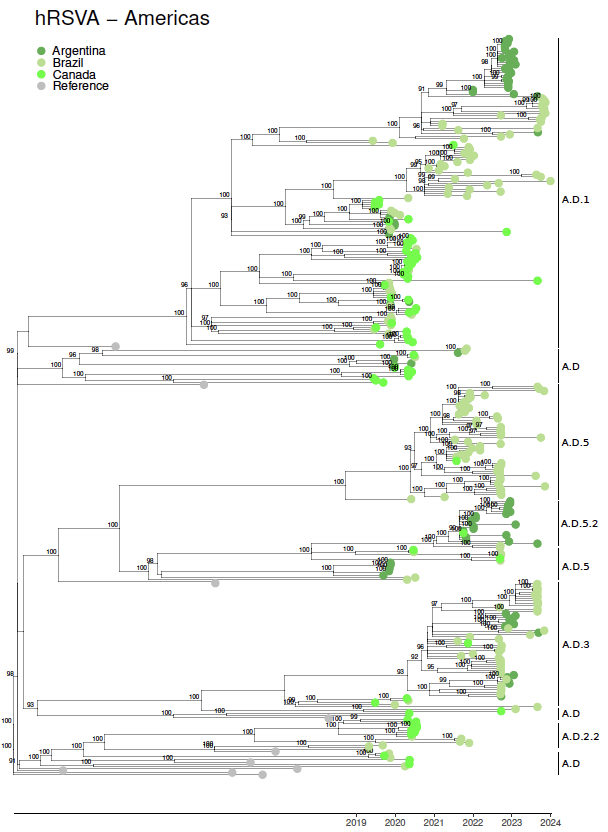


**Supplementary Figure 5A:** Phylogenetic tree of RSV-A sequences from The Americas


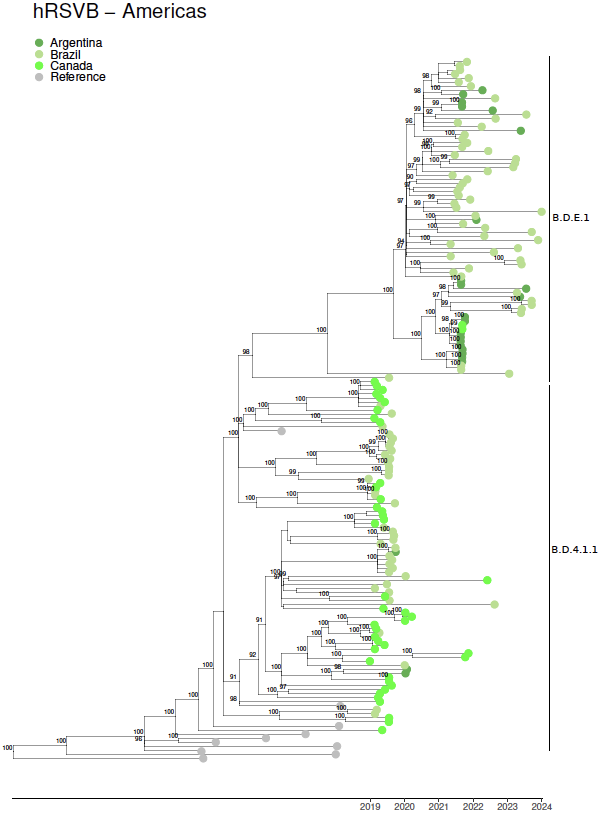


**Supplementary Figure 5B:** Phylogenetic tree of RSV-B sequences from The Americas


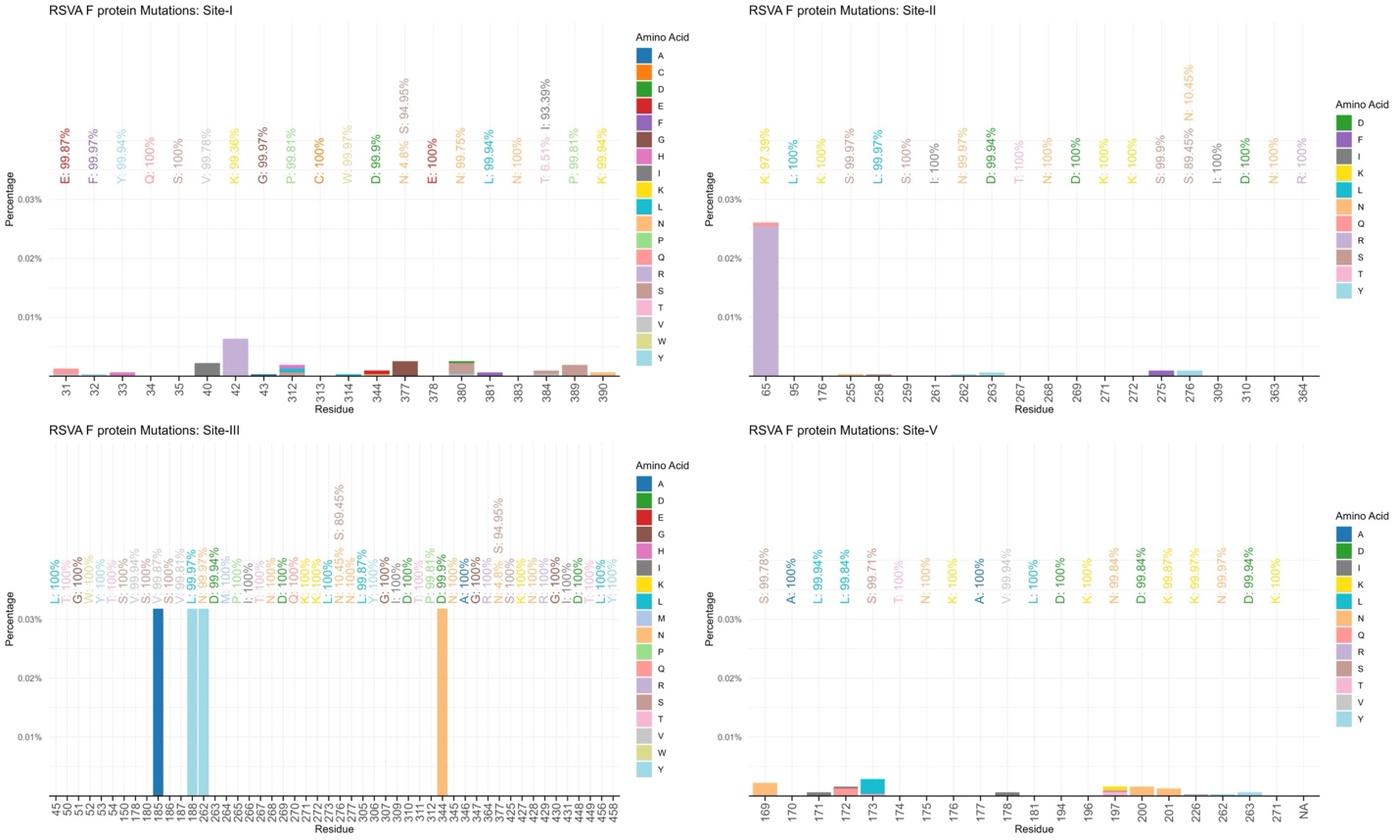


**Supplementary Figure 6A:**  Mutation Analysis of RSV-A F Protein


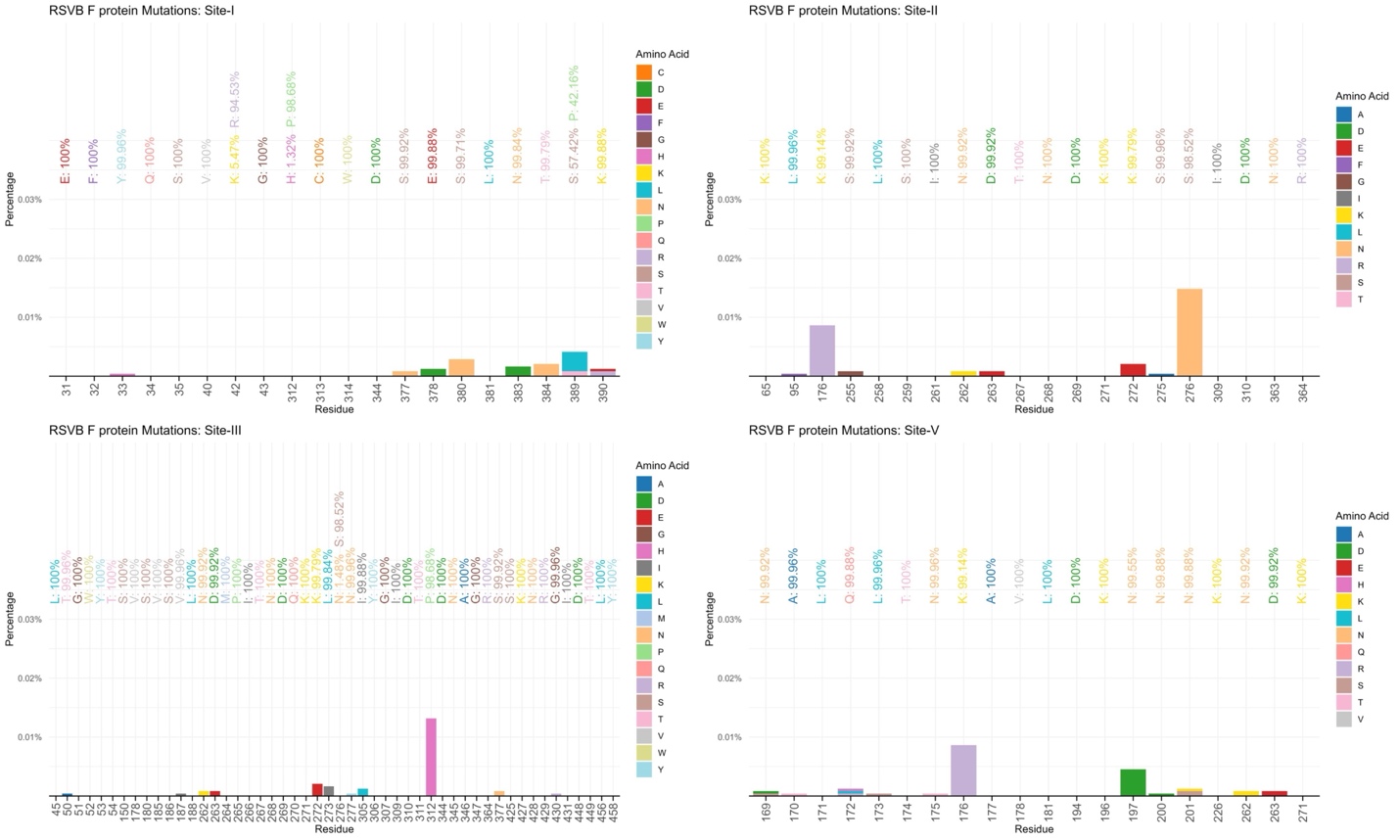


**Supplementary Figure 6B:** Mutation Analysis of RSV-B F Protein
